# IL-15 Is Overexpressed in γδ T Cells and Correlates with Disease Severity in Relapsing-Remitting Multiple Sclerosis

**DOI:** 10.3390/jcm10184174

**Published:** 2021-09-15

**Authors:** Michał K. Zarobkiewicz, Wioleta Kowalska, Izabela Morawska, Paweł Halczuk, Konrad Rejdak, Agnieszka Bojarska-Junak

**Affiliations:** 1 Department of Clinical Immunology, Medical University of Lublin, 20-093 Lublin, Poland; wioleta.kowalska@umlub.pl (W.K.); 51977@student.umlub.pl (I.M.); 2 Department of Neurology, Medical University of Lublin, 20-090 Lublin, Poland; pawelhalczuk@umlub.pl (P.H.); konradrejdak@umlub.pl (K.R.); 3 Department of Histology and Embryology with Experimental Cytology Unit, Medical University of Lublin, 20-080 Lublin, Poland

**Keywords:** IL-15, γδ T cells, multiple sclerosis, MS

## Abstract

Interleukin 15 (IL-15) is known to be involved in the pathogenesis of multiple sclerosis (MS). An animal study revealed a distinct subset of IL-15-producing γδ T cells that correlate with disease severity. The aim of the current study was to test whether such a subset is also present in humans and its importance for the pathogenesis of MS. The peripheral blood from 29 patients with relapsing-remitting MS (including 6 relapses) and 22 controls was stained with monoclonal antibodies and analyzed with flow cytometry. The existence of IL-15+ γδ T cells was confirmed. Moreover, the percentage of IL-15+ γδ T is significantly increased in MS patients and correlates with disease severity. Nevertheless, additional functional studies are needed to fully understand the importance of those cells in multiple sclerosis pathogenesis

## 1. Introduction

Multiple sclerosis is an autoimmune inflammatory demyelinating disease of the central nervous system that affects nearly 2.5 million people worldwide [1]. It is significantly more prevalent among women than men [2]. Apart from health problems, multiple sclerosis exerts a significant economic burden with increased risk for job loss and early pension due to health issues [3]. Indeed, the capacity to work usually decreases with disease duration [4].

**Figure 1:**
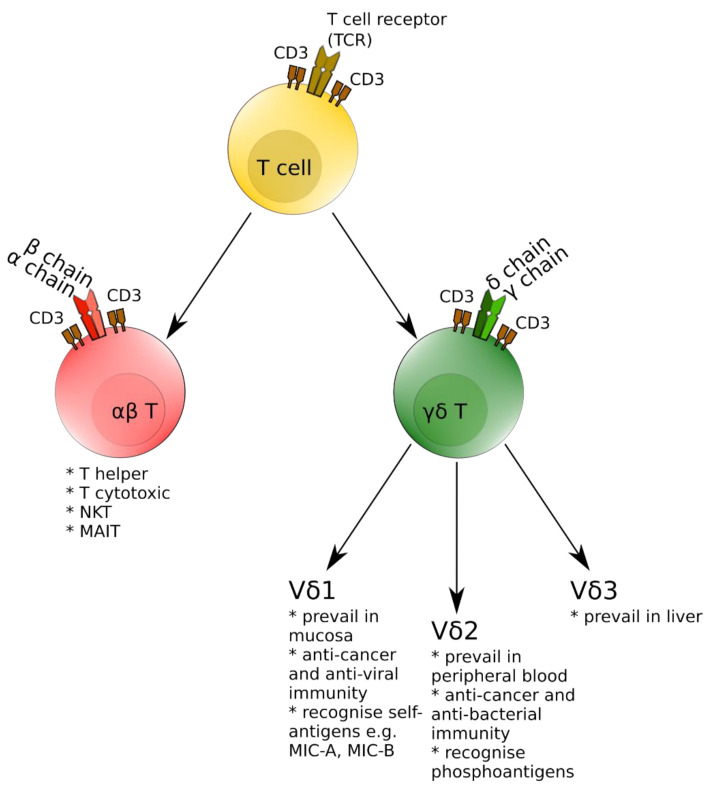
Schematic presentation of major T-cell subsets with special emphasis on γδ T cells.

Along with conventional αβ T cells, γδ T ones comprise a second major T cell subpopulation in humans [5]. The γδ T cells can be further divided into several subsets based on the expression of specific Vγ segments (especially important for rodent γδ T cells) and Vδ segments for human γδ T lymphocytes. In the latter, there are three major subtypes—Vδ1 cells prevailing in mucosal tissues, Vδ2 being the most prevalent subset in peripheral blood and the minor Vδ3 subpopulation, inhabiting mostly the liver [6,7]. γδ T cells are involved in either a positive or negative way in a number of human diseases—from cancer immunosurveillance [8], response to various bacterial and viral infections [9], through involvement in asthma and allergy [10], to autoimmune diseases such as multiple sclerosis (MS) or rheumatoid arthritis [11,12]. For better understanding, Figure 1 presents a simplified version of the T-cell lineage including major subsets with special emphasis on γδ T cells.

Previous studies have suggested the importance of IL-15 in multiple sclerosis pathogenesis [13,14,15]. Although the role of IL-15 in MS pathogenesis is not fully understood, it is suggested that IL-15 may indirectly (via T cytotoxic cells) regulate IL-17A expression by T helper cells [13]. Furthermore, a significant upregulation of IL-15 was observed in relapse, suggesting a possible involvement therein [15]. A recent study on a murine model of MS, experimental autoimmune encephalomyelitis (EAE), revealed a distinct subset of IL-15-producing γδ T cells that promote the expansion of memory T cells, driving the disease further [16]. Thus, we hypothesized that IL-15-producing γδ T cells are also increased in MS patients and that they are involved in MS pathogenesis. Indeed, in the current study, we confirm the existence of IL-15+ γδ T cells among MS patients. Although the percentage of IL-15+ γδ T only weakly correlates with the percentage of memory T cells, it significantly correlates with disease severity.

## 2. Materials and Methods

### 2.1. Study Group

A total of 29 patients (23 remissions and 6 relapses) with the diagnosis of relapsing-remitting multiple sclerosis were recruited for the study at the Department of Neurology, Medical University of Lublin. Additionally, 22 age- and sex-matching healthy adults were included as a control group. Detailed characteristics are presented in Table 1. Inclusion criteria were as in our previous study [17]: I. no other autoimmunological disease diagnosed, II. no glucocorticoid treatment during 4 weeks prior to blood collection, and III. no history of any neoplasm or any neurosurgical treatment. For the control group, the inclusion criteria were as follows: I. were not hospitalized for 6 weeks prior to blood collection, II. were not diagnosed with any autoimmune, neurological or oncologic disease, III. had not undergone neurosurgical treatment in the past, and IV. had no I degree relative with the diagnosis of MS. Each participant gave written informed consent prior to donating 10 mL peripheral blood, taken into an EDTA-containing tube. The majority of subjects were treated with natalizumab (18 out of 26), and the remaining group received either no treatment or one of the following: cladribine, fingolimod, immu-838. The study protocol was approved by the Bioethical Committee at the Medical University of Lublin.

### 2.2. Flow Cytometry

**Figure 2:**
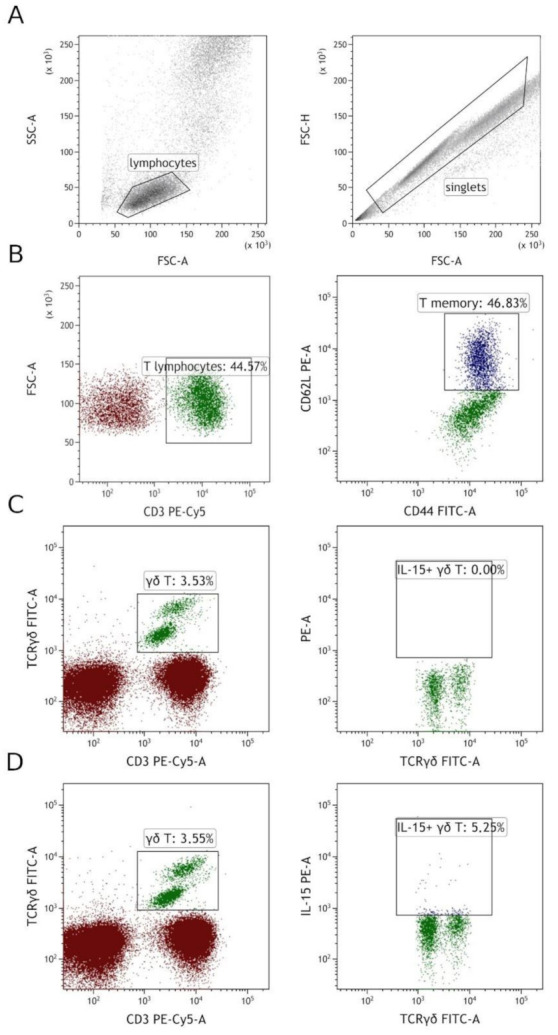
Gating strategy. (**A**) Firstly, singlets were selected based on FSC-H vs. FSC-A, then lymphocytes were gated on SSC vs. FSC. (**B**) T memory cells were gated by first gating CD3+ cells from total lymphocytes, then by gating CD44/CD62L double-positive cells among T cells. (**C**,**D**) γδ T cells were gated on CD3 vs. TCRγδ. Then, among them, IL-15+ cells were gated, panel (**C**) shows FMO (fluorescence minus one) control, and panel (**D**) shows IL-15+ gating on the same patient.

Staining and flow cytometry was performed as previously [17]. First, antibodies for surface staining were added to 100 uL of whole peripheral blood. After 20 min of incubation at room temperature in darkness, erythrocytes were lysed with FACS Lysing Solution (Becton Dickinson, Franklin Lakes, NJ, USA). The FACS Lysing Solution lyses erythrocytes, fixes, and permeabilizes cells [18]. After 10 min of incubation, the sample was centrifuged and supernatant was discarded. Then, 2 mL of PBS was added to the tube and centrifuged to wash out the remaining lysing solution. Next, after the wash, an anti-IL-15 PE antibody was added to γδ T cell tube. As previously, the sample was incubated for 20 min in darkness at room temperature. Next, the excess antibodies were washed out by adding 2 mL of PBS and centrifugation at 700× *g* for 5 min. Finally, samples were resuspended in PBS and acquired using BD FACS Canto II (Becton Dickinson, Franklin Lakes, NJ, USA) and CytoFlex LX (Beckmann Coulter, Brea, CA, USA). The full specifications are presented in Supplementary Table S1. The gating strategy is presented in Figure 2.

The following antibodies were used: anti-CD3 PE-Cy5, anti-TCRγδ FITC, anti-iNKT FITC, anti-CD8 FITC, anti-CD62L PE, anti-CD44 FITC, anti-IL-15 PE, anti-PD-1 PE. Details, including clone and catalog number, are presented in Supplementary Table S2.

### 2.3. Cell Sorting

PBMCs were isolated by gradient centrifugation using Gradisol L (Aqua Medica, Lodz). Isolated PBMCs were stained with monoclonal antibodies anti-CD3 PE-Cy7 and anti-TCRγδ FITC (Supplementary Table S2). After 20 min of incubation in the darkness at room temperature, the excess of antibodies was washed out with PBS. Samples were directly sorted with BD FACS Aria IIu (Becton Dickinson, Franklin Lakes, NJ, USA). The full specification is presented in Supplementary Table S1. Each time after sorting, the purity of cells was assessed. Samples were processed further only if the purity was >95%. Sorted cells immediately after purity assessment were suspended in the RLT buffer (Qiagen, Inc., Valencia, CA, USA) with β-mercaptoethanol and frozen at −80 °C. The gating strategy is presented in Supplementary Figure S2.

### 2.4. RT-qPCR

Finally, total RNA was isolated with QIAamp RNA Blood Mini Kit (Cat No.: 52304; Qiagen, Inc., Valencia, CA, USA), following the manufacturer’s manual. Isolated RNA was stored for a short time at −80 °C. Then, it was transcribed into cDNA with Transcriptor FirstStrand cDNA Synthesis Kit (Roche Applied Science, Mannheim, Germany). Next, a qPCR step was performed with TaqMan Gene Expression Master Mix (Cat No.: 4369016, Thermo Fisher Scientific, Applied Biosystems, Inc., Waltham, MA, USA), using TaqMan probe for IL-15: TaqMan Gene Expression Assay for human IL-15 (Assay ID: Hs01003716_m1). Human ACTB (Beta Actin) Endogenous Control (Applied Biosystems, Austin, TX, USA, 4326315E) was used as endogenous control. Finally, a normalization to β-actin expression was performed using the 2^−ΔCq^ formula. Each sample was run in duplicate. 

### 2.5. Statistical Analysis

Data were analyzed with GraphPad Prism 5 (GraphPad Software, San Diego, CA, USA). Shapiro–Wilk test was used to assess data distribution, and *p* values were calculated with the Kruskal–Wallis test with Dunn correction, only *p* < 0.05 are marked on the graphs and listed in the text. The level of significance was set as *p* < 0.05. All the data are presented with median and interquartile range. Correlations were calculated with the Spearman test. The strength of correlations was assessed as proposed by Chan [19] with ρ correlation coefficient between 0.3 and 0.59 considered fair, ρ between 0.6 and 0.8 strong and ρ > 0.8 as very strong. 

## 3. Results

### 3.1. Human γδ T Express IL-15 and Are Significantly More Prevalent in MS Patients than Healthy Volunteers

**Figure 3:**
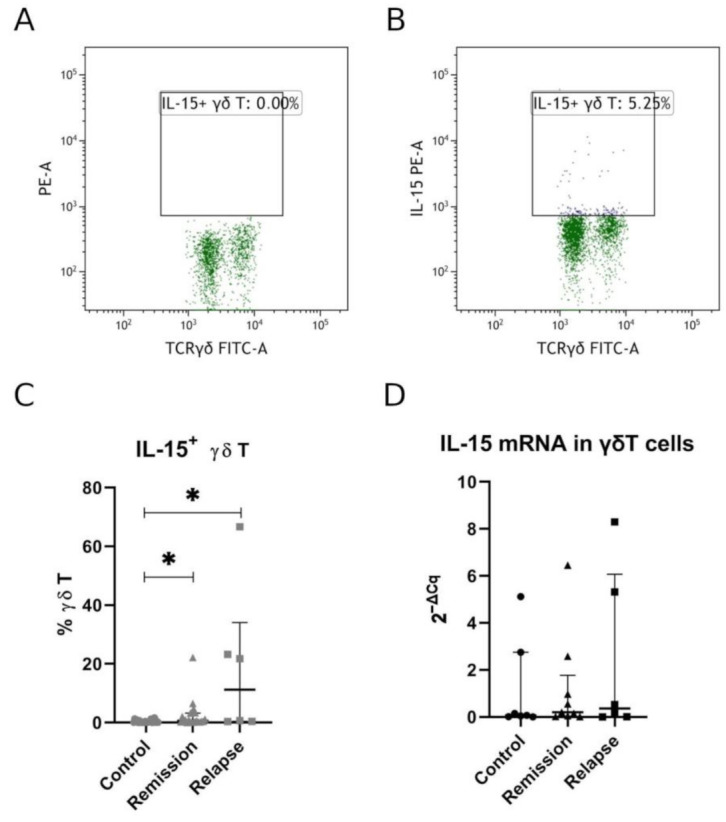
Percentage of IL-15+ γδ T cells in MS patients. Panel (**A**) shows FMO (fluorescence-minus-one) control, and panel (**B**) shows IL-15 staining. Full data set for IL-15 expression measured by flow cytometry is presented in panel(**C**), and panel (**D**) shows relative mRNA expression measured by RT-qPCR. Each shape in panels C and D represents separate patient. Data presented as median, IQR. Statistically significant differences are marked with asterisks (*), relapse vs. control *p* = 0.0096, remission vs. control *p* = 0.0231 (panel (**C**)).

First, we have analyzed the expression of IL-15 in γδ T cells by flow cytometry. Patients in relapse had the highest IL-15+ γδ T percentage. IL-15+ γδ T percentage was significantly higher in both relapse and remission patients when compared to healthy volunteers (relapse vs. control *p* = 0.0096, remission vs. control *p* = 0.0231; Figure 3C). No significant difference was, however, observed for relapse vs. remission comparison. 

We have also analyzed the expression of IL-15 by αβT cells by gating on CD3+ and TCRγδ- events. Remission showed a clear but not significant tendency toward higher percentages, and relapse showed significant increase in IL-15+ cells compared to control group (Supplementary Figure S2).

### 3.2. IL-15 Expression by γδ T Was Confirmed by RT-qPCR

To further confirm that IL-15 observed in flow cytometry was expressed by γδ T cells themselves, we have sorted γδ T cells to perform RT-qPCR. Here, we have confirmed a low level of IL-15 expression with a tendency to higher expression among MS patients (Figure 3D). This lack of significance could probably be attributed to limited group sizes for RT-qPCR.

### 3.3. T Memory Cell Percentage Was Insignificantly Higher among MS Patients

**Figure 4:**
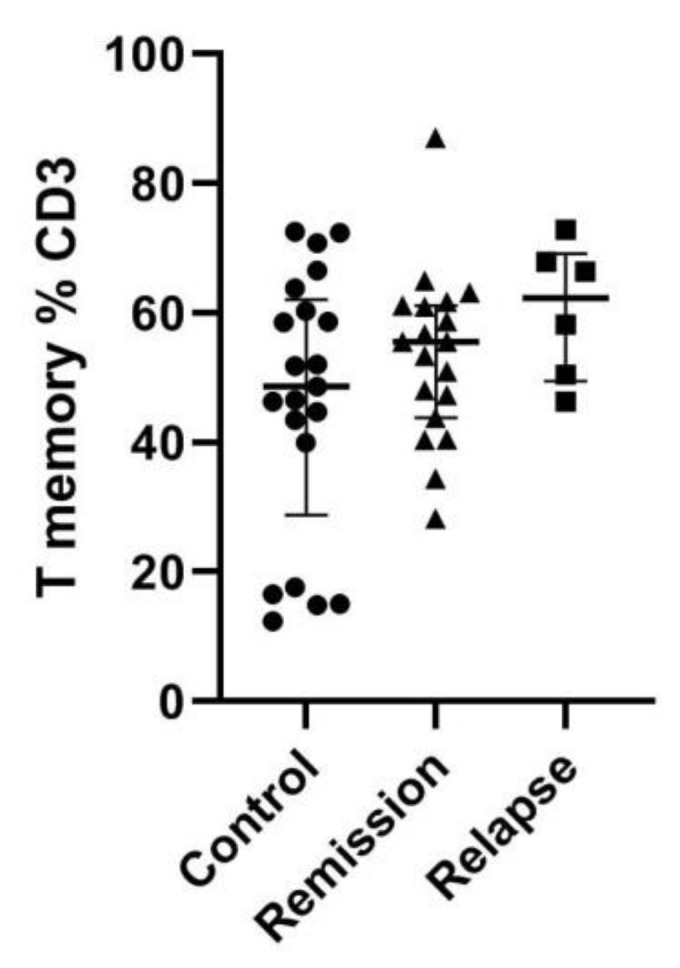
Memory T-cell percentage among total T cells. Memory cells were gated as CD62L+/CD44+. Data presented as median and IQR.

Next, we have assessed the percentage of T memory cells as in the initial study on the murine model [16]. There is a clear tendency toward higher percentages among MS patients, especially during relapse. That trend, however, did not reach statistical significance (Figure 4). 

### 3.4. IL-15 Expressing γδ T Cells Positively Correlate with Disease Severity in MS

Finally, we have analyzed the correlation of IL-15 expressing γδ T cells with a set of parameters in MS patients—both clinical and immunological. Data for iNKT and Tc percentages and expression of PD-1 on iNKT and CD8+ T cells were collected for an ongoing study that partially used blood from the same patients—thus, these data are used here only for correlations. 

**Figure 5:**
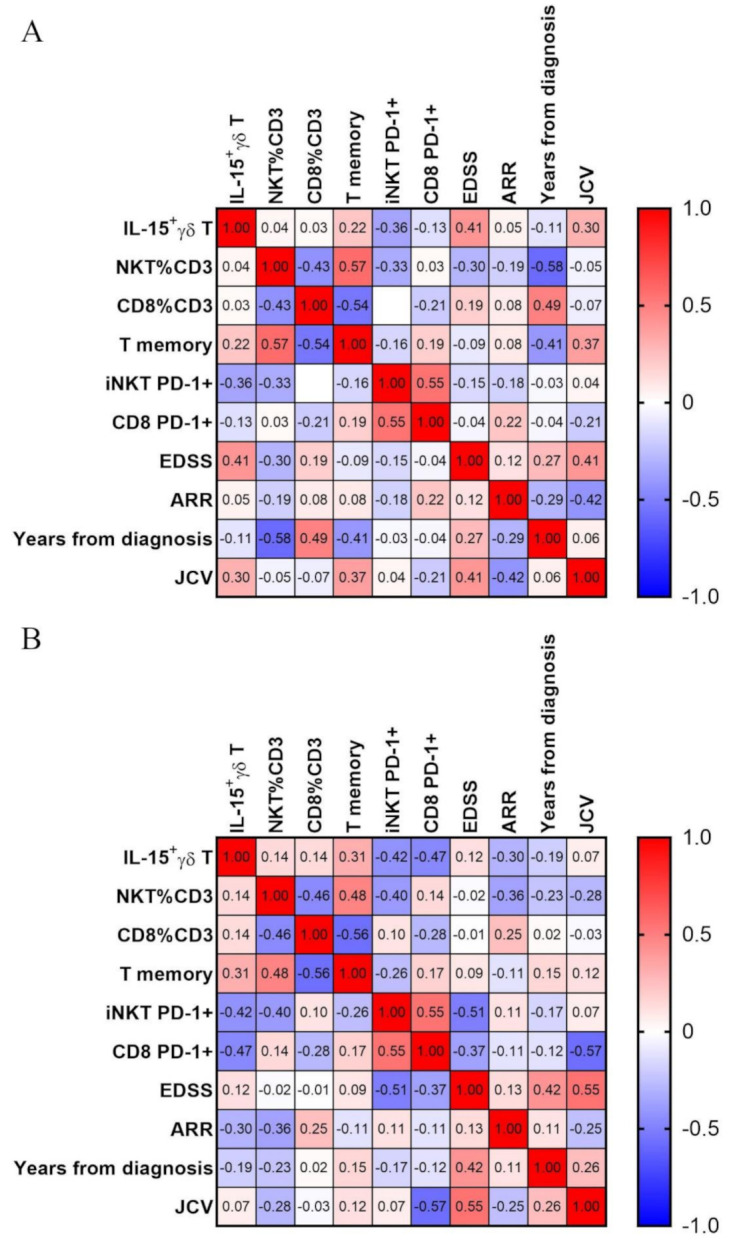
Correlation matrix. Correlations were calculated with Spearman test, and ⍴ values for each correlation are presented in respective squares. Panel (**A**) shows all MS patients, panel (**B**) only those in remission.

A significant, positive correlation was observed between IL-15+ γδ T percentage and disease severity, measured as EDSS (extended disease severity score) (Figure 5). Correlation with EDSS has only fair strength (ρ = 0.41). On the other hand, there was no correlation with the T memory subset. Surprisingly, the T memory subset correlates negatively with disease duration (years from diagnosis, ρ = −0.41).

We then next analyzed the correlation after exclusion of all relapse patients. In this setting, there is no real correlation with EDSS; there is however one with T memory percentage (ρ = 0.31) and stronger negative correlation with percentages of iNKT (ρ = −0.42) and CD8+ T cells (ρ = −0.47). Finally, the percentage of IL-15+ gdT cells correlate weakly with the second measure of disease activity—ARR (annual relapse rate) with ρ = −0.3.

## 4. Discussion

In the current study, we have proved the existence of IL-15+ γδ T cells in humans. Moreover, we have shown that they are more prevalent in MS patients and that they correlate with disease severity (EDSS). 

In line with the results of Wang et al., we have shown that IL-15+ γδ T cells are up-regulated in MS patients compared to healthy controls [16]. Pashenkov et al. observed an elevation of IL-15+ PBMC in MS patients compared to healthy volunteers—still, this was only observed for chronic progressive MS, not for RRMS [20]. Which agrees with our results regarding αβT cells. Pashenkov et al. also noted that IL-15+ PBMCs correlate with disease severity and disability [20], similarly to our observation with IL-15+ γδ T.

IL-15 is one of the common-γ- chain cytokines along with IL-2, IL-4, IL-7, IL-9, and IL-21 [21]. B cells and monocytes seem to be two major sources of IL-15 in multiple sclerosis patients [22]. Interestingly, IL-15 knockout mice develop a significantly more severe course of EAE [23], and IL-15 treatment can lessen the EAE severity [24]. On the other hand, administration of IL-15 during the acute phase, shortly after EAE onset, worsens the symptoms [25]. Moreover, IL-15 is significantly overexpressed in mononuclear cells in both peripheral blood and, to an even greater extent, in cerebrospinal fluid of MS patients [14]. It is also increased in serum of MS patients during relapse, but no difference was observed between stable MS patients and healthy controls [15]. 

IL-15 signaling is dependent on so-called transpresentation—in order to be active IL-15 has to be coupled with IL-15Rɑ, only such a complex can be recognized by IL-15Rβ and leads to signal transduction [21]. Thus, IL-15 seems to act in the close vicinity with its source cell. Surface expression of IL-15 on monocytes and B cells is twice as frequent in MS patients compared to healthy volunteers [22]. IL-15 is important not only for cytotoxic ɑβ T but also for NK, iNKT and γδ T cells survival, proliferation and cytotoxic activity [21]. Interestingly, we haven’t observed any correlation between percentages of IL-15+ γδ T cells and iNKT or CD8+ T cells. One could expect an accumulation of cytotoxic cells when IL-15 production is increased.

IL-15 also promotes IL-17 production by Th cells (to a significantly greater extent in MS patients than in healthy individuals) [25]. IL-15 promotes cytotoxic T-cells migration through the blood–brain barrier into the central nervous system, granzyme B production, as well as cytotoxicity against oligodendrocytes [22]. What is even more important, up to 90% of astrocytes in MS plaques are expressing IL-15 while they rarely do that in normal brain tissue [26]. Moreover, cytotoxic T lymphocytes are usually in close proximity to IL-15+ astrocytes [26]. Similarly to conventional cytotoxic T lymphocytes, IL-15 promotes cytotoxicity of Vδ2 γδ T cells with increased expression of perforin, granulysin, and granzyme B [27]. IL-15 is also upregulated during MS relapses [28]. All that can at least partially explain our observation that IL-15+ γδ T cells correlated positively with disability (EDSS). 

The current study has several limitations. First of all, it is made on a limited sample size, especially for relapse. Moreover, not all patients are treatment free, thus complicating the image. In fact the majority of patients in the current study were treated with natalizumab, which is known for significantly altering the lymphocyte count (most significantly rising the number of B and NK cells, to a lesser extent T cells); fortunately, the CD4/CD8 ratio is preserved, suggesting that natalizumab-driven accumulation of lymphocytes is uniform for all T subsets [29]. Finally, we have used unconventional markers for T memory cells—CD44. It is a well-studied marker for mouse T memory cells; nevertheless, it has been previously shown to be also a reliable marker for human memory T cells [30]. Thus, the results of the current study should be confirmed in a separate study on a cohort of treatment-naive patients. 

The available data suggest that IL-15 has an important proinflammatory role and that its increase promotes demyelination and disease progression. The current study shows a novel property of γδ T cells in general and its possible implication for MS pathogenesis. γδ T cells require IL-2 or IL-15 for their survival, proliferation, and effector functions, but they do not produce IL-2 by themselves. In the current study, we have shown that γδ T can produce IL-15. Therefore, they may be able to promote their own survival and function under certain circumstances which may have further implications for MS. 

## 5. Conclusions

In the current paper, we have shown that IL-15+ γδ T cells are present in human peripheral blood and their percentage is significantly increased in multiple sclerosis patients. Most importantly, IL-15+ γδ T cells correlate positively with disease severity.

## Figures and Tables

**Table 1:** Basic clinical and sociodemographic data about study participants. Data are presented as median, IQR. EDSS—expanded disability status scale, ARR—annual relapse rate.

Group	% Women	Age	EDSS	ARR	Years from Diagnosis
MS	68	39 (30.5–48)	3.5 (3–5.75)	0 (0–1)	10 (4–14.5)
Healthy	65	48 (26–58)	n/a	n/a	n/a

## Data Availability

The data presented in this study are available on request from the corresponding author. The data are not publicly available due to patient protection.

## Supplementary Materials

The following are available online at https://www.mdpi.com/article/10.3390/jcm10184174/s1, Figure S1: Gating strategy for cell sorting, Figure S2: Percentage of IL-15+ cells among αβ T cells, Table S1: The detailed configuration of cytometers, Table S2: List of all antibodies used in the current study.

## References

[1] Browne P., Chandraratna D., Angood C., Tremlett H., Baker C., Taylor B.V., Thompson A.J. (2014). Atlas of Multiple Sclerosis 2013: A Growing Global Problem with Widespread Inequity. Neurology.

[2] Hunter S.F. (2016). Overview and Diagnosis of Multiple Sclerosis. Am. J. Manag. Care.

[3] Hilt Pfleger C.C., Meulengracht Flachs E., Koch-Henriksen N. (2010). Social Consequences of Multiple Sclerosis (1): Early Pension and Temporary Unemployment—A Historical Prospective Cohort Study. Mult. Scler. J..

[4] Valadkeviciene D., Jatuzis D., Kizlaitiene R., Zukauskaite I., Venceviciene L. (2021). Working Capacity Level of Patients with Multiple Sclerosis in Lithuania: Its Dynamics and Relationship with the Employment and Lethal Outcomes. Mult. Scler. Relat. Disord..

[5] Kabelitz D., Lettau M., Janssen O. (2017). Immunosurveillance by Human Γδ T Lymphocytes: The Emerging Role of Butyrophilins. F1000Research.

[6] Pang D.J., Neves J.F., Sumaria N., Pennington D.J. (2012). Understanding the Complexity of Γδ T-Cell Subsets in Mouse and Human. Immunology.

[7] Kenna T., Golden-Mason L., Norris S., Hegarty J.E., O’Farrelly C., Doherty D.G. (2004). Distinct Subpopulations of Gamma Delta T Cells Are Present in Normal and Tumor-Bearing Human Liver. Clin. Immunol..

[8] Clark B.L., Thomas P.G. (2020). A Cell for the Ages: Human Γδ t Cells across the Lifespan. Int. J. Mol. Sci..

[9] Zheng J., Liu Y., Lau Y.-L., Tu W. (2013). Γδ-T Cells: An Unpolished Sword in Human Anti-Infection Immunity. Cell. Mol. Immunol..

[10] Zarobkiewicz M.K., Wawryk-Gawda E., Kowalska W., Janiszewska M., Bojarska-Junak A. (2021). Γδ T Lymphocytes in Asthma: A Complicated Picture. Arch. Immunol. Ther. Exp..

[11] Zarobkiewicz M.K., Kowalska W., Roliński J., Bojarska-Junak A.A. (2019). Γδ T Lymphocytes in the Pathogenesis of Multiple Sclerosis and Experimental Autoimmune Encephalomyelitis. J. Neuroimmunol..

[12] Bank I. (2020). The Role of Gamma Delta T Cells in Autoimmune Rheumatic Diseases. Cells.

[13] Yu P., Bamford R.N., Waldmann T.A. (2014). IL-15-Dependent CD8 + CD122 + T Cells Ameliorate Experimental Autoimmune Encephalomyelitis by Modulating IL-17 Production by CD4 + T Cells: Immunomodulation. Eur. J. Immunol..

[14] Kivisäkk P., Matusevicius D., He B., Söderström M., Fredrikson S., Link H. (1998). IL-15 MRNA Expression Is up-Regulated in Blood and Cerebrospinal Fluid Mononuclear Cells in Multiple Sclerosis (MS). Clin. Exp. Immunol..

[15] Rentzos M., Nikolaou C., Rombos A., Evangelopoulos M.E., Dimitrakopoulos A., Kararizou E., Koutsis G., Zoga M., Tsoutsou A., Sfangos K. (2010). Circulating Interleukin-15 and RANTES Chemokine in MS Patients: Effect of Treatment with Methylprednisolone in Patients with Relapse. Neurol. Res..

[16] Wang X., Wei Y., Liu X., Xing C., Han G., Chen G., Hou C., Dambuza I.M., Shen B., Li Y. (2015). IL-15-Secreting γδ T Cells Induce Memory T Cells in Experimental Allergic Encephalomyelitis (EAE) Mice. Mol. Immunol..

[17] Zarobkiewicz M.K., Kowalska W., Halczuk P., Woś J., Jodłowska-Jędrych B., Rejdak K., Roliński J., Bojarska-Junak A.A. (2019). RORγT Is Overexpressed in INKT and Γδ T Cells during Relapse in Relapsing-Remitting Multiple Sclerosis. J. Neuroimmunol..

[18] Bekkema R., Tadema A., Daenen S.M.G.J., Kluin-Nelemans H.C., Mulder A.B. (2008). An Improved Flow Cytometric Method Using FACS Lysing Solution for Measurement of ZAP-70 Expression in B-Cell Chronic Lymphocytic Leukemia. Cytom. B Clin. Cytom..

[19] Chan Y.H. (2003). Biostatistics 104: Correlational Analysis. Singapore Med. J..

[20] Pashenkov M., Mustafa M., Kivisäkk P., Link H. (1999). Levels of Interleukin-15-Expressing Blood Mononuclear Cells Are Elevated in Multiple Sclerosis. Scand. J. Immunol..

[21] Allard-Chamard H., Mishra H.K., Nandi M., Mayhue M., Menendez A., Ilangumaran S., Ramanathan S. (2020). Interleukin-15 in Autoimmunity. Cytokine.

[22] Schneider R., Mohebiany A.N., Ifergan I., Beauseigle D., Duquette P., Prat A., Arbour N. (2011). B Cell-Derived IL-15 Enhances CD8 T Cell Cytotoxicity and Is Increased in Multiple Sclerosis Patients. J. Immunol..

[23] Gomez-Nicola D., Spagnolo A., Guaza C., Nieto-Sampedro M. (2010). Aggravated Experimental Autoimmune Encephalomyelitis in IL-15 Knockout Mice. Exp. Neurol..

[24] Wu X., Pan W., He Y., Hsuchou H., Kastin A.J. (2010). Cerebral Interleukin-15 Shows Upregulation and Beneficial Effects in Experimental Autoimmune Encephalomyelitis. J. Neuroimmunol..

[25] Laurent C., Deblois G., Clénet M.-L., Moratalla A.C., Farzam-kia N., Girard M., Duquette P., Prat A., Larochelle C., Arbour N. (2021). Interleukin-15 Enhances Proinflammatory T-Cell Responses in Patients with MS and EAE. Neurol. Neuroimmunol. Neuroinflamm..

[26] Saikali P., Antel J.P., Pittet C.L., Newcombe J., Arbour N. (2010). Contribution of Astrocyte-Derived IL-15 to CD8 T Cell Effector Functions in Multiple Sclerosis. J. Immunol..

[27] Aehnlich P., Carnaz Simões A.M., Skadborg S.K., Holmen Olofsson G., thor Straten P. (2020). Expansion With IL-15 Increases Cytotoxicity of Vγ9Vδ2 T Cells and Is Associated With Higher Levels of Cytotoxic Molecules and T-Bet. Front. Immunol..

[28] Blanco-Jerez C., Plaza J.F., Masjuan J., Orensanz L.M., Alvarez-Cermeno J.C. (2002). Increased Levels of IL-15 MRNA in Relapsing–Remitting Multiple Sclerosis Attacks. J. Neuroimmunol..

[29] Khoy K., Mariotte D., Defer G., Petit G., Toutirais O., Le Mauff B. (2020). Natalizumab in Multiple Sclerosis Treatment: From Biological Effects to Immune Monitoring. Front. Immunol..

[30] Zhou J., Nagarkatti P., Zhong Y., Nagarkatti M. (2011). Characterization of T-Cell Memory Phenotype after in Vitro Expansion of Tumor-Infiltrating Lymphocytes from Melanoma Patients. Anticancer Res..

