# Supplementary material for: IL-15 Is Overexpressed in γδ T Cells and Correlates with Disease Severity in Relapsing-Remitting Multiple Sclerosis
Source: J Clin Med. 2021 Sep 15;10(18):4174. doi: 10.3390/jcm10184174 (PMC8467081; doi:10.3390/jcm10184174)

Supplementary table S1. The detailed configuration of cytometers

BD FACS Canto II			
No	Laser	Filter setup	Detector name
1	405nm	450/50	Pacific Blue
2		510/50	AmCyan
3	488nm	530/30	FITC
4		585/42	PE
5		670LP mirror	PerCp-Cy5.5
6		780/60	PE-Cy7
7	633nm	660/20	APC
8		780/60	APC-Cy7
BD FACS Aria IIu			
		Filter setup	Detector name
1	488nm	530/30	FITC
2		576/26	PE
3		610/20	PE-TexasRed
4		695/40	PerCp-Cy5.5
5		780/60	PE-Cy7
6	633nm	660/20	APC
7		780/60	APC-Cy7
Beckman Coulter Cytoflex LX			
No	Laser	Filter setup	Detector name
1	375nm	450/45	NUV450

2		525/40	NUV525
3		675/30	NUV675
4	405nm	450/45	Pacific Blue
5		525/40	Krome Orange
6		610/20	V610
7		660/10	V660
8		763/43	V763
9	488nm	525/40	FITC
10		610/20	ECD
11		690/50	PE-Cy5.5
12	561nm	585/42	PE
13		610/20	mCherry
14		675/30	PE-Cy5
15		710/50	PE-Cy5.5
16		763/43	PE-Cy7
17	638nm	660/10	APC
18		712/25	APC-A700
19		763/43	APC-A750
20	808nm	840/20	Alexa Fluor 790
21		885/40	IR885

Supplementary table S2. List of all antibodies used in the current study. All antibodies are specific for human antigens

Name	Fluorochrome	Clone	Manufacturer, cat. nr
anti-CD3	PE-Cy5	HIT3a	BD, 555341
anti-TCRγδ	FITC	B1	BioLegend, 331208
anti-iNKT	FITC	6B11	BioLegend, 342906
anti-CD62L	PE	DREG-56	BioLegend, 304806
anti-CD44	FITC	BJ18	BioLegend, 338804
anti-IL-15	PE	34559	R&D, IC2471P
anti-CD8	FITC	RPA-T8	BD, 555366
anti-PD-1	PE	EH12.1	BD, 560795
anti-CD3	PE-Cy7	UCHT1	BD, 563423

Supplementary figure S1. Gating strategy for cell sorting (**panel A**), after sort purity (**panel B**). The percentage of γδT in B panel is among total events.

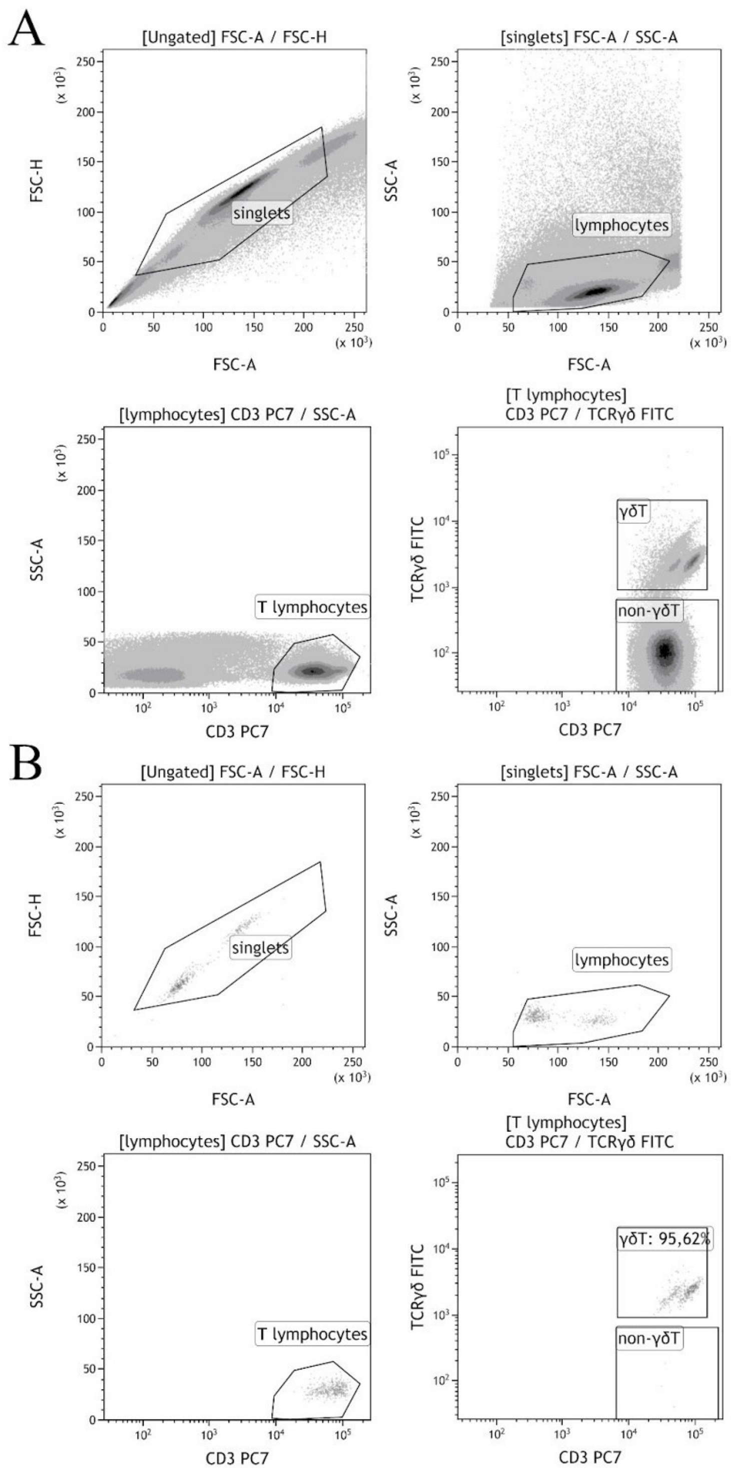

Supplementary figure S2. IL-15 expression in αβ T cells (gated as CD3+/TCRγδ-). Significant overexpression was noted in relapse patients and visible tendency towards increased expression can be noted for remission patients. Data presented with median and IQR.

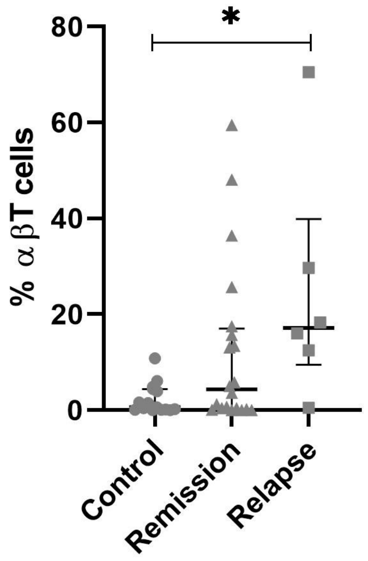

Supplement: Supplementary file 1 [file jcm-10-04174-s001.zip › jcm-1359632-supplementary.pdf]
